# Association between depressive symptoms and pericardial fat in healthy older men and women

**DOI:** 10.1038/s41598-022-17888-4

**Published:** 2022-08-17

**Authors:** Natalie Ella Miller, Andrew Steptoe

**Affiliations:** Department of Behavioural Science and Health, Institute of Epidemiology and Health Care, University College London, 1-19 Torrington Place, Gower Street, London, UK

**Keywords:** Cardiovascular diseases, Predictive markers, Risk factors, Cardiovascular diseases

## Abstract

Depressive symptoms are associated with increased risk for cardiovascular disease (CVD), and inflammation may contribute to this relationship. Pericardial fat, a highly metabolically active fat depot, is implicated in the pathogenesis of CVD, but its association with depressive symptoms is unclear. This study examined the cross-sectional and longitudinal association between depressive symptoms and pericardial fat over a three-year period. Participants were 543 healthy men and women (mean age = 62.9 years) without history or objective signs of coronary heart disease from the Whitehall II cohort. In men, depressive symptoms were positively associated with pericardial fat at baseline after adjustment for sociodemographics, waist to hip ratio and conventional cardiovascular risk factors. Inflammation, indexed by plasma interleukin 6 concentration, accounted for 17% of this association. Longitudinally, depressive symptoms did not predict pericardial fat three years later in men once baseline levels of pericardial fat were accounted for. No significant associations between depressive symptoms and pericardial fat were found in women. Overall, our findings suggest that greater pericardial fat might be a mechanism by which depressive symptoms are associated with increased risk for CVD in men, and inflammation may also lie on this pathway.

## Introduction

Cardiovascular disease (CVD) remains a major cause of death, disability and rising healthcare costs worldwide^1^. A large body of cohort studies have revealed a positive association between depressive symptoms and CVD incidence in initially healthy populations^2,3^. Studies have also shown that the severity of depression may interact with triglyceride level to further increase CVD risk^4^. Many mechanisms have been proposed to underly the association between depressive symptoms and CVD incidence, such as dysregulation in the hypothalamic–pituitary–adrenal axis, elevated inflammation, endothelial dysfunction, and altered platelet activation^5–7^. Understanding the mechanisms that may underly the link between depressive symptoms and CVD is important as this can inform the design of both preventive and therapeutic interventions.

Obesity is a well-established risk factor for CVD^8^. The association between depression and adiposity has been studied extensively, and some investigators have championed the “Jolly Fat hypothesis” which postulates that obesity is associated with lower depression^9–11^. Nevertheless, evidence for the “Jolly Fat hypothesis” is mixed, and some studies have found that obesity may be associated with higher depression^12^. One potential explanation for these inconsistent findings is that the use of body mass index (BMI) to measure obesity is limited as it does not distinguish between fat and lean mass, and does not take into account fat distribution^13^. Another issue is heterogeneity among depressive symptoms in relation to adiposity. A recent analysis of more than 57,000 individuals aged from 18 to 100 years showed consistent positive associations between greater BMI and depression, with strong links with a subset of depressive symptoms^14^. These symptom-specific associations were confirmed in longitudinal analysis of 122,341 participants in UK Biobank.

Pericardial fat is a unique fat depot that has been associated with many CVD risk factors, markers of atherosclerosis and adverse cardiac events, independent of overall adiposity^15–18^. Pericardial fat might be implicated in the pathogenesis of CVD due to its proximity to the coronary arteries and shared microcirculation with the myocardium, meaning that any factors released by pericardial fat can readily interact with the adjacent cardiomyocytes^19,20^. Like intra-abdominal adipose tissue, pericardial fat is highly metabolically active and secretes pro-inflammatory cytokines such as interleukin-6 (IL-6) and tumor necrosis factor-α (TNF-α)^21^. Thus, it is hypothesised that pericardial fat leads to a local inflammatory response that may contribute to the development of CVD^18^.

Two small-scale studies from Hannover, Germany have reported that patients with diagnosed major depressive disorder (MDD) have greater volumes of pericardial fat than individuals without MDD^22,23^. However, these studies are limited as they did not control statistically for central adiposity, a correlate of pericardial fat^16^. Thus, it is unclear whether individuals with MDD have greater volumes of pericardial fat than individuals without MDD due to their depression status, or due to greater levels of central adiposity. Furthermore, to the author’s knowledge, no study has examined the association between depressive symptoms and pericardial fat volume in the general population. Therefore, the aim of this study was to examine cross-sectional and prospective associations between depressive symptoms and pericardial fat over a three-year period in individuals without history or objective signs of coronary heart disease (CHD) from the Whitehall II cohort. All analyses were sex-stratified due to established sex differences in depressive symptoms and pericardial fat^22–24^, and were adjusted statistically for central adiposity. We also examined whether inflammation, indexed by IL-6, is a mediator of the association between depressive symptoms and pericardial fat. We reasoned that because inflammation is thought to contribute to the association between depression and CVD and is positively correlated with both depressive symptoms and pericardial fat^6,25,26^, it may be a biological pathway linking depressive symptoms and pericardial fat.

## Methods

### Participants

The participants in this study were drawn from the Heart Scan Study, which is part of the Whitehall II study^27^. The Whitehall II study was a prospective cohort study of 10,308 British civil servants which investigated demographic, psychosocial and biological risk factors for CVD. The Heart Scan Study recruited a sub-sample of healthy participants from the Whitehall II cohort between 2006–2008 and investigated the psychobiological pathways linking psychosocial factors and risk for CVD^28,29^. Participants in this sub-sample were of White European origin, were aged between 53–76 years, had no history or objective signs of CHD, and had no previous diagnosis or treatment for hypertension, diabetes, inflammatory diseases, or allergies. The sampling method for this study was stratified by employment grade, which acted as a marker for socioeconomic status. This sampling method resulted in an appropriate representation of individuals from lower, intermediate and higher grades of employment. All participants provided full informed written consent to participate in the study and ethical approval was obtained from the University College London Hospital committee on the Ethics of Human Research. All methods were performed in accordance with relevant guidelines and regulations.

### Measures

#### Pericardial fat

Pericardial fat was assessed at baseline and three-year follow up using methods that have been described previously^30^. In brief, noncontrast coronary artery calcium scans were taken using an electron beam computed tomography scanner (GE-Imatron C-150; GE-Imatron, San Francisco, CA). Volume of pericardial fat (in cm^3^) was calculated by two experienced investigators blinded to any psychophysiological and clinical data on a Siemens multimodality workstation (Siemens, Forchheim, Germany). To account for differences in heart size, we normalised pericardial fat volume by body surface area (giving values in cm^3^/m^2^) and used these values in analyses^31^.

#### Depressive symptoms

Depressive symptoms were assessed at baseline and three-year follow up using the Centre for Epidemiological Studies Depression Scale (CES-D)^32^. The CES-D consists of 20 items which assess the frequency of depressive symptoms during the past week on a four-point Likert scale. The total CES-D score ranges from 0 to 60, with higher scores indicating greater depression.

#### Covariates

Demographic covariates included age and grade of employment. Current smoking levels were reported by participants. Because pericardial fat is associated with greater central adiposity^16^, we controlled statistically for waist to hip ratio. Waist circumference was measured by an experienced nurse midway between the lowest rib and iliac crest and hip circumference at the level of the great trochanters for the calculation of waist to hip ratio. Blood samples were taken either after an eight hour overnight fast or at least a four hour fast after a light, fat-free breakfast. Serum for lipid analyses was stored at 4 °C, and high-density lipoprotein (HDL) cholesterol and triglycerides were measured within 72 h using enzymatic colorimetric methods. Concentration of low-density lipoprotein (LDL) cholesterol was calculated using the Friedewald equation^33^. Plasma IL-6 concentration was measured using a high-sensitivity enzyme-linked immunosorbent assay (ELISA) (R & D Systems, Oxford, UK). The homeostasis model assessment index for insulin resistance (HOMA-IR) was calculated as fasting serum insulin (μIU/mL) × fasting serum glucose (mmol/L)/22.5^34^. Systolic blood pressure was measured three times while seated using an automated UA-779 digital monitor. An average was computed from the second and third values.

### Statistical analysis

Descriptive statistics for participants’ baseline characteristics were calculated separately for men and women. Triglycerides, IL-6 and HOMA-IR were positively skewed so were log transformed prior to analyses. Sex differences in baseline characteristics were assessed using the *t* test for continuous variables and the χ^2^ test for categorical variables.

Data were missing at random for 0–4.8% for different covariates, so the primary analyses were carried out following imputation. Multiple imputation by chained equations was used to impute missing data on the covariates^35^. Twenty datasets were imputed, and the results combined using Rubin’s rules^36,37^. The following variables were used as predictors in the multiple imputation model: demographic factors (age, sex, employment grade), health factors (waist circumference, hip circumference, smoking) and biological factors (LDL cholesterol, HDL cholesterol, triglycerides, systolic blood pressure, IL-6, fasting serum insulin, fasting serum glucose). The observed and imputed data were largely similar, indicating that the multiple imputation procedure achieved its goals (Supplementary Table 1).

Multivariable linear regression analyses were performed to assess (1) the cross-sectional association between depressive symptoms and pericardial fat; (2) the prospective association between depressive symptoms and pericardial fat three years later; and (3) the involvement of IL-6 in the association between depressive symptoms and pericardial fat. In the cross-sectional analyses, model 1 adjusted for age and grade of employment; model 2 additionally adjusted for waist to hip ratio to control for central adiposity and isolate the effect of depressive symptoms on pericardial fat; and model 3 further adjusted for smoking, LDL cholesterol, HDL cholesterol, triglycerides, systolic blood pressure and HOMA-IR. Results are presented as unstandardised regression coefficients (*B*) with corresponding 95% confidence intervals (CIs), standard errors (SEs) and *p* values. In the longitudinal analyses, the first three models were the same as in the cross-sectional analyses, however, model 4 included baseline levels of pericardial fat to examine the association between depressive symptoms and changes in pericardial fat over time. To examine the involvement of IL-6 in the association between depressive symptoms and pericardial fat, IL-6 was included as an additional covariate to the fully adjusted models. The change in *B* following adjustment for IL-6 was taken to indicate the role of inflammation in explaining the relationship between depressive symptoms and pericardial fat^38^. All analyses were sex-stratified. Variance inflation factors were below 10 for all regression models and tolerance values were greater than 0.2, indicating that multicollinearity was not present. Robust standard errors were calculated when heteroscedasticity was present within regression models^39^.

Two sensitivity analyses were carried out. Firstly, all analyses were ran on participants without missing data on any variable included in the analysis. Second, all analyses were repeated using raw values of pericardial fat volume instead of pericardial fat volume normalised by body surface area. Analyses were performed using Stata Statistical Software Version 17^40^.

## Results

### Descriptive statistics

The sample characteristics of men and women in the observed data are presented in Table 1. In total, the study sample comprised 543 individuals, of which 294 were men and 249 were women. Compared to men, women had lower volumes of pericardial fat adjusted for body surface area (*p* < 0.001). There were no sex differences in depressive symptoms measured by the CES-D (*p* > 0.05).

**Table 1:** Participant characteristics at baseline, stratified by sex (observed data).

Variable	Males (*N* = 280–294)		Females (*N* = 188–249)		*P* difference
	*N*	Mean (SD)/*n* (%)	*N*	Mean (SD)/*n* (%)	
Age	294	62.04 (5.72)	249	63.89 (5.41)	< 0.001
Grade of employment	294		249		0.011
Lower		52 (17.69)		70 (28.11)	
Intermediate		126 (42.86)		86 (34.54)	
Higher		116 (39.46)		93 (37.35)	
Smoking status	294		249		0.927
Non-smoker		278 (94.56)		235 (94.38)	
Smoker		16 (5.44)		14 (5.62)	
Waist circumference (cm)	288	92.69 (10.49)	249	79.86 (11.69)	< 0.001
Hip circumference (cm)	288	98.46 (6.96)	249	98.47 (8.95)	0.988
Waist to hip ratio	288	0.94 (0.07)	249	0.81 (0.08)	< 0.001
LDL cholesterol (mmol/L)	280	2.88 (0.81)	244	3.17 (0.89)	< 0.001
HDL cholesterol (mmol/L)	283	1.51 (0.38)	245	1.86 (0.49)	< 0.001
Triglycerides (mmol/L)^a^	283	1.56 (0.94)	244	1.19 (0.59)	< 0.001
Systolic blood pressure (mmHg)	288	127.92 (13.57)	240	123.94 (15.92)	0.002
Fasting glucose (mmol/L)	287	5.09 (0.51)	242	5.00 (0.78)	0.105
Fasting insulin (μIU/mL)	285	7.07 (5.27)	243	6.04 (6.48)	0.046
HOMA-IR^a^	285	1.64 (1.49)	242	1.38 (1.98)	0.086
Interleukin-6 (pg/mL)^a^	287	1.37 (0.84)	238	1.30 (0.81)	0.232
Depressive symptoms total score (CES-D)	294	6.32 (6.16)	248	7.05 (6.90)	0.196
Pericardial fat adjusted for body surface area (cm^3^/m^2^)	285	66.45 (24.47)	188	55.44 (18.75)	< 0.001

^a^Variable was log transformed before analysis.

*SD* standard deviation, *HDL* high-density lipoprotein, *HOMA-IR* insulin resistance, *LDL* low-density lipoprotein.

### Association between depressive symptoms and pericardial fat

In men, depressive symptoms were significantly associated with pericardial fat at baseline after adjustment for age and employment grade (*B* = 0.618, 95% CI 0.136–1.100, *p* = 0.012, Model 1) (Table 2). The association between depressive symptoms and pericardial fat in men remained statistically significant after adjustment for waist to hip ratio (*B* = 0.590, 95% CI 0.160–1.020, *p* = 0.007, Model 2). Further adjustment for smoking, LDL cholesterol, HDL cholesterol, triglycerides, systolic blood pressure and HOMA-IR had little effect on this association (*B* = 0.512, 95% CI 0.099–0.925, *p* = 0.015, Model 3). However, additional adjustment for IL-6 attenuated the association by 17% (*B* = 0.425, 95% CI 0.027–0.824, *p* = 0.037, Model 4). The full details of the final regression results are presented in Supplementary Table 2. Other independent predictors of pericardial fat in men included waist to hip ratio, HDL cholesterol, HOMA-IR and IL-6. In women, there was no statistically significant association between depressive symptoms and pericardial fat at baseline in any of the models. IL-6 was also not associated with pericardial fat at baseline (*B* = 9.515, 95% CI − 14.340–33.371, *p* = 0.432). The only independent predictors of pericardial fat in women were age and waist to hip ratio.

**Table 2:** Regression coefficients showing cross-sectional associations between depressive symptoms and pericardial fat adjusted for body surface area.

	Males (*N* = 285)			Females (*N* = 187)		
	*B* (95% CI)	SE	*P* value	*B* (95% CI)	SE	*P* value
Model 1	0.618 (0.136, 1.100)	0.245	0.012*	0.225 (− 0.216, 0.666)	0.224	0.315
Model 2	0.590 (0.160, 1.020)	0.219	0.007**	0.091 (− 0.341, 0.523)	0.219	0.678
Model 3	0.512 (0.099, 0.925)	0.210	0.015*	0.135 (− 0.315, 0.585)	0.228	0.555
Model 4	0.425 (0.027, 0.824)	0.202	0.037*	0.119 (− 0.334, 0.571)	0.229	0.606

SE = robust standard errors to adjust for heteroscedasticity.

*p < 0.05, **p < 0.01, ***p < 0.001.

Model 1 adjusted for age and grade of employment. Model 2 adjusted for age, grade of employment and waist to hip ratio. Model 3 adjusted for age, grade of employment, waist to hip ratio, smoking, LDL cholesterol, HDL cholesterol, triglycerides, systolic blood pressure and HOMA-IR. Model 4 adjusted for age, grade of employment, waist to hip ratio, smoking, LDL cholesterol, HDL cholesterol, triglycerides, systolic blood pressure, HOMA-IR and IL-6.

*CI* confidence interval, *HDL* high-density lipoprotein, *HOMA-IR* insulin resistance, *LDL* low-density lipoprotein, *IL-6* interleukin-6.

The longitudinal associations between depressive symptoms and pericardial fat are presented in Table 3. In men, depressive symptoms were associated with pericardial fat three years later, after adjustment for age and employment grade (*B* = 0.610, 95% CI 0.111–1.109, *p* = 0.017, Model 1). This association remained statistically significant after further adjustment for waist to hip ratio (*B* = 0.584, 95% CI 0.129–1.038, *p* = 0.012, Model 2). Additional adjustment for smoking, LDL cholesterol, HDL cholesterol, triglycerides, systolic blood pressure and HOMA-IR had little effect on the association (*B* = 0.525, 95% CI 0.102–0.948, *p* = 0.015, Model 3). However, after adjustment for baseline levels of pericardial fat, the association between depressive symptoms and pericardial fat three years later in men was attenuated (*B* = 0.117, 95% CI − 0.177–0.410, *p* = 0.435, Model 4). The longitudinal association between depressive symptoms and pericardial fat remained non-significant after further adjustment for IL-6 (Model 5). In women, there was no association between depressive symptoms and pericardial fat three years later in any of the models. The full regression results for depressive symptoms predicting pericardial fat three years later in men and women are presented in Supplementary Table 3. The only independent predictors of future pericardial fat in men were waist to hip ratio, HOMA-IR and baseline levels of pericardial fat. In women, the only independent predictor of future pericardial fat was baseline levels of pericardial fat.

**Table 3:** Longitudinal associations between depressive symptoms and pericardial fat adjusted for body surface area.

	Males (*N* = 269)			Females (*N* = 180)		
	*B* (95% CI)	SE	*P* value	*B* (95% CI)	SE	*P* value
Model 1	0.610 (0.111, 1.109)	0.253	0.017*	0.106 (− 0.337, 0.548)	0.224	0.638
Model 2	0.584 (0.129, 1.038)	0.231	0.012*	0.007 (− 0.399, 0.413)	0.206	0.972
Model 3	0.525 (0.102, 0.948)	0.215	0.015*	0.042 (− 0.373, 0.456)	0.210	0.843
Model 4	0.117 (− 0.177, 0.410)	0.149	0.435	− 0.097 (− 0.267, 0.074)	0.086	0.263
Model 5	0.113 (− 0.180, 0.407)	0.149	0.447	− 0.087 (− 0.268, 0.094)	0.092	0.342

SE = robust standard errors to adjust for heteroscedasticity.

*p < 0.05, **p < 0.01, ***p < 0.001.

Model 1 adjusted for age and grade of employment. Model 2 adjusted for age, grade of employment and waist to hip ratio. Model 3 adjusted for age, grade of employment, waist to hip ratio, smoking, LDL cholesterol, HDL cholesterol, triglycerides, systolic blood pressure and HOMA-IR. Model 4 adjusted for age, grade of employment, waist to hip ratio, smoking, LDL cholesterol, HDL cholesterol, triglycerides, systolic blood pressure, HOMA-IR and baseline levels of pericardial fat. Model 5 adjusted for age, grade of employment, waist to hip ratio, smoking, LDL cholesterol, HDL cholesterol, triglycerides, systolic blood pressure, HOMA-IR, baseline levels of pericardial fat and IL-6.

*CI* confidence interval, *HDL* high-density lipoprotein, *HOMA-IR* insulin resistance, *LDL* low-density lipoprotein, *IL-6* interleukin-6.

### Sensitivity analyses

Complete case analyses yielded similar results to the multiple imputed datasets; however, depressive symptoms were not associated with pericardial fat three years later in men after adjustment for baseline levels of pericardial fat (Supplementary Tables 5, 6). Analyses using raw values of pericardial fat volume yielded similar results to analyses using pericardial fat volume adjusted for body surface area (Supplementary Tables 6, 7).

## Discussion

In this study of middle-aged and older healthy men and women, depressive symptoms were cross-sectionally associated with pericardial fat in men, but not women. The cross-sectional association between depressive symptoms and pericardial fat in men remained significant after adjusting for central adiposity (indexed by waist to hip ratio) and CVD risk factors, suggesting that the association is independent of these factors. Inclusion of IL-6 concentration reduced the association by 17%, suggesting that inflammation partly accounted for the relationship. Longitudinally, depressive symptoms predicted pericardial fat three years later in men, but not after baseline levels of pericardial fat were accounted for. The longitudinal results suggest that depressive symptoms are not associated with changes in pericardial fat over a 3-year time period.

As expected, we found a sex-specific relationship between depressive symptoms and pericardial fat at baseline. This finding is in line with many studies showing sex differences in the association between depression and visceral adipose tissue^41–43^. For example, a cross-sectional study of 1581 women and 1718 men from the Framingham Heart Study found that depressive symptoms were positively associated with visceral adipose tissue after adjustment for overall obesity and other confounders in women, but not men^43^. However, prior studies focus on intra-abdominal adipose tissue as a type of visceral fat. The present study demonstrates a sex-specific relationship between depressive symptoms and pericardial fat, a unique fat depot surrounding the heart. Indeed, there are known sex differences in pericardial fat accumulation. For instance, many studies show that men have more pericardial fat than women, in line with the findings of the present study^22,23,44^. There are also known sex differences in depressive symptoms, with women generally presenting with greater depressive symptoms than men^45^, although in our study, we found no sex differences in depressive symptoms. One potential reason for this discrepancy is that the sample of this study were drawn from a healthy white-collar population, and thus many of the psychosocial factors that might predict sex differences in depression have been accounted for (e.g., health status, employment status)^46^.

The mechanisms underlying the sex-specific association between depressive symptoms and pericardial fat are unclear. Inflammation only explained 17% of the association between depressive symptoms and pericardial fat in men, suggesting that other factors must also play a role. A meta-analysis of genome-wide association studies has found several single nucleotide polymorphisms (SNPs) associated with pericardial fat^47^. A longitudinal study has shown that these SNPs are associated with change in pericardial fat over time, independent of sex^48^. However, it is unknown whether there are genetic variants that have sex-specific effects on pericardial fat and its association with depression. Future research is needed to explore whether there are genetic variants that contribute to the sex-specific association between depressive symptoms and pericardial fat. Alternatively, it is possible that lifestyle factors play a role in this sex-specific association. Physical activity has been shown to be inversely associated with pericardial fat, independent of overall adiposity^30^. Improved diet quality has been associated with a reduction in pericardial fat over time, independent of multiple demographic and lifestyle covariates^48^. However, evidence suggests that there might be sex differences in physical activity and dietary patterns, such that women tend to be less active but have better diet quality than men^49,50^. Thus, future longitudinal research could explore whether diet quality and physical activity contribute to the sex-specific relationship between depressive symptoms and pericardial fat, and whether these lifestyle factors interact with genetic risk for pericardial fat in the sex-specific relationship between depressive symptoms and pericardial fat.

The present study found further sex differences in cardiovascular risk factors related to pericardial fat. Plasma IL-6 was associated with pericardial fat in men but not women once sociodemographics, central adiposity, cardiovascular risk factors and depressive symptoms had been taken into account. Furthermore, HOMA-IR was also associated with pericardial fat in men but not women once these covariates had been taken into account. Other studies have shown that pericardial fat is associated with IL-6^21^ and insulin resistance^51,52^. However, no studies have shown that these associations are sex-specific. One potential explanation for these sex-specific associations is that sexual dimorphism in pericardial fat may occur with ageing. Experimental data have revealed that pericardial fat function decreases with ageing in female rats but not in male rats^53^. These findings may explain why there are sex differences in the relationship of pericardial fat with IL-6 and insulin resistance in middle-aged and older men and women. Another sex difference found in the present study related to pericardial fat is that it was positively associated with HDL cholesterol in men but not in women. This finding is not in line with prior studies which have found negative associations between pericardial fat and HDL cholesterol in men and women^16,54^. A final sex difference related to pericardial fat in the present study is that age was a significant independent predictor of pericardial fat at baseline in women, but not men. This finding is in line with evidence showing that women accumulate visceral fat at a greater rate and intensity during the ageing process than men^55^. One potential reason for this sex difference is the role of menopause and menopause-related changes in sex hormones. Post-menopausal women have been shown to have greater volumes of pericardial fat than pre-menopausal women, and it is thought that declines in oestrogen contribute to these increased volumes^56^.

We also found that depressive symptoms were associated with pericardial fat three years after depression assessment in men, but not once baseline levels of pericardial fat were accounted for. This suggests that the observed prospective association was a reflection of the cross-sectional results, and that depressive symptoms were not associated with changes in pericardial fat in men over time. Many longitudinal studies have revealed an association between depressive symptoms and other visceral fat depots such as abdominal fat, independent of overall adiposity^57–59^. However, it has been argued that unlike other visceral fat depots, pericardial fat does not respond to small changes in body weight^60^. Some studies have shown that weight loss by lifestyle intervention (exercise/diet) and bariatric surgery can reduce pericardial fat, but most of these were conducted in participants with obesity^60,61^. Thus, it is possible that obesity is causally linked to pericardial fat accumulation, and that obesity may moderate the prospective association between depressive symptoms and pericardial fat. Alternatively, it is possible that changes in pericardial fat may depend on body composition and fat distribution. Intervention studies have found that changes in pericardial fat with weight loss are associated with initial levels and changes in visceral abdominal fat^62,63^. In our study, we found that waist to hip ratio was an independent predictor of changes in pericardial fat in men. Taken together, these findings suggest that changes in pericardial fat in men may depend on initial levels and changes in abdominal fat. Future work exploring the moderating role of abdominal and general obesity on the prospective relationship between depressive symptoms and pericardial fat in men is warranted.

This study has several strengths. It was carried out in a large, well characterised population in which detailed cardiovascular measures have been monitored for several years, increasing confidence that this was a healthy cohort of older men and women. Depressive symptoms were assessed with an established, validated measure, and we included a range of sociodemographic and health covariates. The sampling method for the sample was stratified to include a broad range of socioeconomic status, and participants were excluded if they had history or objective signs of CVD, previous diagnosis or treatment for hypertension, diabetes, inflammatory diseases, or allergies. Thus, any observed associations were not confounded by differences in health status. The present study also has some limitations. All participants were of White European background, which limits generalisation to other ethnic groups; this issue is particularly pertinent given there are ethnic differences in lifestyle factors such as diet quality, physical activity, smoking and alcohol use, as well as obesity^64^, which may contribute to ethnic differences in pericardial fat volume and its association with CVD^65^. Second, given that participants were older than 50 and ageing can affect pericardial fat function, the findings may not be generalisable to younger adults^66^. Future longitudinal work is needed to examine the association between depressive symptoms and pericardial fat in younger adults. Third, the study is observational, so might be prone to residual confounding by unmeasured factors and thus causal conclusions cannot be made. Fourth, the follow-up period of this study was only 3 years which might have been too short to demonstrate a longitudinal association between depressive symptoms and changes in pericardial fat. Finally, this study only examined one cytokine, IL-6, as a potential mediator of the association between depressive symptoms and pericardial fat. Future studies could examine other inflammatory markers as potential mediators of this association such as interleukin-1 beta (IL-1β) which has been associated with both depression^67^ and pericardial fat^68^.

To conclude, this study has shown that greater pericardial fat might be a mechanism linking depressive symptoms and increased risk of CVD in men, and inflammation may also lie on this pathway. These findings suggest that interventions designed to reduce pericardial fat and depressive symptoms might help reduce risk of CVD in men. Ketogenic diets are thought to have antidepressant^69^ and fat reducing effects^70^, and thus might be promising interventions, although no randomised controlled trials have yet been published. A protective role for Mediterranean diets has also been proposed^71^. Future research exploring other biological and behavioural factors that may contribute to the association between pericardial fat and depressive symptoms in men could help further our understanding of how depressive symptoms are linked to CVD.

## Supplementary Information

Supplementary Information.

## Data Availability

Whitehall II data are available to *bona fide* researchers for research purposes. The Whitehall II data sharing policy can be found at https://www.ucl.ac.uk/epidemiology-health-care/research/epidemiology-and-public-health/research/whitehall-ii/data-sharing.
