# Supplementary material for: Association between depressive symptoms and pericardial fat in healthy older men and women
Source: Sci Rep. 2022 Aug 17;12:13959. doi: 10.1038/s41598-022-17888-4 (PMC9385858; doi:10.1038/s41598-022-17888-4)
Supplement: Supplementary file 1 — Supplementary Information. [file 41598_2022_17888_MOESM1_ESM.docx]

**Supplementary Table 1.** Comparison of observed and imputed data at baseline, stratified by sex (page 2)

**Supplementary Table 2.** Results of regression analysis for depressive symptoms predicting pericardial fat at baseline (page 4)

**Supplementary Table 3.** Results of regression analysis for depressive symptoms predicting pericardial fat three years later (page 6)

**Supplementary Table 4.** Cross-sectional associations between depressive symptoms and pericardial fat adjusted for body surface area (complete case analysis) (page 8)

**Supplementary Table 5.** Longitudinal associations between depressive symptoms and pericardial fat adjusted for body surface area (complete case analysis) (page 9)

**Supplementary Table 6.** Cross-sectional associations between depressive symptoms and raw values of pericardial fat volume (page 11)

**Supplementary Table 7.** Longitudinal associations between depressive symptoms and raw values of pericardial fat volume (page 12)

**Supplementary Table 1.** Comparison of observed and imputed data at baseline, stratified by sex.

|  | Observed data | | | | Imputed data | | | |
| --- | --- | --- | --- | --- | --- | --- | --- | --- |
|  | Males  (*N* = 280-294) | | Females  (*N* = 188-249) | | Males  (*N* = 294) | | Females  (*N* = 249) | |
| Variable | *N* | Mean (SD)/*n* (%) | *N* | Mean (SD)/*n* (%) | *N* | Mean (SD)/*n* (%) | *N* | Mean (SD)/*n* (%) |
| Age | 294 | 62.04 (5.72) | 249 | 63.89 (5.41) | 294 | 62.04 (5.72) | 249 | 63.89 (5.41) |
| Grade of employment | 294 |  | 249 |  | 294 |  | 249 |  |
| Lower |  | 52 (17.69) |  | 70 (28.11) |  | 52 (17.69) |  | 70 (28.11) |
| Intermediate |  | 126 (42.86) |  | 86 (34.54) |  | 126 (42.86) |  | 86 (34.54) |
| Higher |  | 116 (39.46) |  | 93 (37.35) |  | 116 (39.46) |  | 93 (37.35) |
| Smoking status | 294 |  | 249 |  | 294 |  | 249 |  |
| Non-smoker |  | 278 (94.56) |  | 235 (94.38) |  | 278 (94.56) |  | 235 (94.38) |
| Smoker |  | 16 (5.44) |  | 14 (5.62) |  | 16 (5.44) |  | 14 (5.62) |
| Waist circumference (cm) | 288 | 92.69 (10.49) | 249 | 79.86 (11.69) | 294 | 92.74 (10.56) | 249 | 79.86 (11.69) |
| Hip circumference (cm) | 288 | 98.46 (6.96) | 249 | 98.47 (8.95) | 294 | 98.50 (7.02) | 249 | 98.47 (8.95) |
| Waist to hip ratio | 288 | 0.94 (0.07) | 249 | 0.81 (0.08) | 294 | 0.94 (0.07) | 249 | 0.81 (0.08) |
| LDL cholesterol (mmol/L) | 280 | 2.88 (0.81) | 244 | 3.17 (0.89) | 294 | 2.88 (0.85) | 249 | 3.17 (0.90) |
| HDL cholesterol (mmol/L) | 283 | 1.51 (0.38) | 245 | 1.86 (0.49) | 294 | 1.51 (0.39) | 249 | 1.86 (0.50) |
| Triglycerides (mmol/L)* | 283 | 1.56 (0.94) | 244 | 1.19 (0.59) | 294 | 1.57 (0.97) | 249 | 1.20 (0.61) |
| Systolic blood pressure (mmHg) | 288 | 127.92 (13.57) | 240 | 123.94 (15.92) | 294 | 127.91 (13.63) | 249 | 124.02 (16.09) |
| Fasting glucose (mmol/L) | 287 | 5.09 (0.51) | 242 | 5.00 (0.78) | 294 |  | 249 |  |
| Fasting insulin (μIU/mL) | 285 | 7.07 (5.27) | 243 | 6.04 (6.48) | 294 |  | 249 |  |
| HOMA-IR* | 285 | 1.64 (1.49) | 242 | 1.38 (1.98) | 294 | 1.64 (1.49) | 249 | 1.45 (2.46) |
| Interleukin-6 (pg/mL)* | 287 | 1.37 (0.84) | 238 | 1.30 (0.81) | 294 | 1.37 (0.85) | 249 | 1.30 (0.83) |
| Depressive symptoms total score (CES-D) | 294 | 6.32 (6.16) | 248 | 7.05 (6.90) | 294 | 6.32 (6.16) | 248 | 7.05 (6.90) |
| Pericardial fat adjusted for body surface area (cm^3^/m^2^) | 285 | 66.45 (24.47) | 188 | 55.44 (18.75) | 285 | 66.45 (24.47) | 188 | 55.44 (18.75) |

*Variable was log transformed before analysis

HDL, high-density lipoprotein; HOMA-IR, insulin resistance; LDL, low-density lipoprotein; SD, standard deviation.

**Supplementary Table 2.** Results of regression analysis for depressive symptoms predicting pericardial fat at baseline

|  | Males (*N* = 285) | | | Females (*N* = 187) | | |
| --- | --- | --- | --- | --- | --- | --- |
|  | *B* (95% CI) | SE | *P* value | *B* (95% CI) | SE | *P* value |
| Age | 0.275 (-0.177, 0.727) | 0.230 | 0.232 | 0.784 (0.236, 1.332) | 0.278 | 0.005** |
| Grade of employment |  |  |  |  |  |  |
| Lower | Ref |  |  | Ref |  |  |
| Intermediate | 2.982 (-3.261, 9.225) | 3.171 | 0.348 | -6.413 (-12.992, 0.166) | 3.333 | 0.056 |
| Higher | 0.595 (-5.709, 6.900) | 3.202 | 0.853 | -6.138 (-12.618, 0.342) | 3.283 | 0.063 |
| Smoking status |  |  |  |  |  |  |
| Non-smoker | Ref |  |  | Ref |  |  |
| Smoker | 5.211 (-4.832, 15.254) | 5.101 | 0.308 | 4.774 (-9.551, 19.099) | 7.257 | 0.512 |
| Waist to hip ratio | 125.798 (71.791, 179.804) | 27.430 | <0.001*** | 47.533 (15.866, 79.199) | 16.043 | 0.003** |
| LDL cholesterol | 2.633 (-0.280, 5.546) | 1.479 | 0.076 | -1.769 (-4.976, 1.438) | 1.624 | 0.278 |
| HDL cholesterol | 7.517 (0.110, 14.924) | 3.762 | 0.047* | -1.765 (-7.337, 3.806) | 2.823 | 0.533 |
| Triglycerides | 12.599 (-11.100, 36.298) | 12.035 | 0.296 | 22.389 (-7.863, 52.641) | 15.324 | 0.146 |
| Systolic blood pressure | -0.027 (-0.211, 0.157) | 0.094 | 0.775 | -0.073 (-0.262, 0.115) | 0.095 | 0.442 |
| Depressive symptoms | 0.425 (0.027, 0.824) | 0.202 | 0.037* | 0.119 (-0.334, 0.571) | 0.229 | 0.606 |
| HOMA-IR | 39.470 (18.993, 59.946) | 10.399 | <0.001*** | 7.626 (-6.937, 22.189) | 7.377 | 0.303 |
| IL-6 | 35.778 (16.228, 55.328) | 9.928 | <0.001*** | 9.515 (-14.340, 33.371) | 12.085 | 0.432 |

Note. SE = robust standard errors to adjust for heteroscedasticity

*p<.05, **p<.01, ***p<.001

CI, confidence interval; HDL, high-density lipoprotein; HOMA-IR, insulin resistance; LDL, low-density lipoprotein; IL-6, interleukin-6.

**Supplementary Table 3.** Results of regression analysis for depressive symptoms predicting pericardial fat three years later

|  | Males (*N* = 269) | | | Females (*N* = 180) | | |
| --- | --- | --- | --- | --- | --- | --- |
|  | *B* (95% CI) | SE | *P* value | *B* (95% CI) | SE | *P* value |
| Age | 0.059 (-0.258, 0.377) | 0.161 | 0.712 | 0.198 (-0.131, 0.528) | 0.167 | 0.236 |
| Grade of employment |  |  |  |  |  |  |
| Lower | Ref |  |  | Ref |  |  |
| Intermediate | -1.463 (-5.951, 3.025) | 2.279 | 0.521 | 3.334 (-0.837, 7.504) | 2.112 | 0.116 |
| Higher | -3.374 (-7.969, 1.222) | 2.333 | 0.149 | 1.485 (-2.055, 5.025) | 1.793 | 0.409 |
| Smoking status |  |  |  |  |  |  |
| Non-smoker | Ref |  |  | Ref |  |  |
| Smoker | 6.145 (-1.669, 13.960) | 3.968 | 0.123 | -0.192 (-4.973, 4.588) | 2.421 | 0.936 |
| Waist to hip ratio | 37.297 (11.869, 62.725) | 12.909 | 0.004** | 18.914 (-0.221, 38.049) | 9.691 | 0.053 |
| LDL cholesterol | 1.959 (-0.419, 4.337) | 1.207 | 0.106 | 1.058 (-0.701, 2.817) | 0.891 | 0.237 |
| HDL cholesterol | 0.550 (-4.534, 5.634) | 2.581 | 0.831 | -0.053 (-3.541, 3.436) | 1.767 | 0.976 |
| Triglycerides | -3.553 (-19.283, 12.177) | 7.985 | 0.657 | -6.185 (-22.845, 10.475) | 8.436 | 0.465 |
| Systolic blood pressure | 0.039 (-0.072, 0.150) | 0.056 | 0.494 | 0.016 (-0.093, 0.125) | 0.055 | 0.767 |
| HOMA-IR | 13.162 (0.484, 25.841) | 6.437 | 0.042* | 8.559 (-3.593, 20.711) | 6.154 | 0.166 |
| Baseline levels of pericardial fat | 0.776 (0.660, 0.892) | 0.059 | <0.001*** | 0.805 (0.704, 0.907) | 0.051 | <0.001*** |
| Depressive symptoms | 0.113 (-0.180, 0.407) | 0.149 | 0.447 | -0.087 (-0.268, 0.094) | 0.092 | 0.342 |
| IL-6 | 1.748 (-13.417, 16.913) | 7.699 | 0.821 | -3.889 (-17.134, 9.357) | 6.707 | 0.563 |

Note. SE = robust standard errors to adjust for heteroscedasticity

*p<.05, **p<.01, ***p<.001

CI, confidence interval; HDL, high-density lipoprotein; HOMA-IR, insulin resistance; LDL, low-density lipoprotein; IL-6, interleukin-6.

**Supplementary Table 4.** Cross-sectional associations between depressive symptoms and pericardial fat adjusted for body surface area (complete case analysis)

|  | Males (*N* = 251) | | | Females (*N* = 169) | | |
| --- | --- | --- | --- | --- | --- | --- |
|  | *B* (95% CI) | SE | *P* value | *B* (95% CI) | SE | *P* value |
| Model 1 | 0.641 (0.129, 1.152) | 0.260 | 0.014* | 0.176 (-0.278, 0.629) | 0.230 | 0.445 |
| Model 2 | 0.646 (0.187, 1.105) | 0.233 | 0.006** | 0.039 (-0.407, 0.486) | 0.226 | 0.862 |
| Model 3 | 0.584 (0.136, 1.032) | 0.228 | 0.011* | 0.078 (-0.381, 0.536) | 0.232 | 0.739 |
| Model 4 | 0.497 (0.067, 0.927) | 0.218 | 0.024* | 0.057 (-0.401, 0.516) | 0.232 | 0.806 |

Note. SE = robust standard errors to adjust for heteroscedasticity

*p<.05, **p<.01, ***p<.001

Model 1 adjusted for age and grade of employment. Model 2 adjusted for age, grade of employment and waist to hip ratio. Model 3 adjusted for age, grade of employment, waist to hip ratio, smoking, LDL cholesterol, HDL cholesterol, triglycerides, systolic blood pressure and HOMA-IR. Model 4 adjusted for age, grade of employment, waist to hip ratio, smoking, LDL cholesterol, HDL cholesterol, triglycerides, systolic blood pressure, HOMA-IR and IL-6.

CI, confidence interval; HDL, high-density lipoprotein; HOMA-IR, insulin resistance; LDL, low-density lipoprotein; IL-6, interleukin-6.

**Supplementary Table 5.** Longitudinal associations between depressive symptoms and pericardial fat adjusted for body surface area (complete case analysis)

|  | Males (*N* = 238) | | | | | Females (*N* = 163) | | |
| --- | --- | --- | --- | --- | --- | --- | --- | --- |
|  | *B* (95% CI) | SE | | | *P* value | *B* (95% CI) | SE | *P* value |
| Model 1 | 0.597 (0.078, 1.115) | | 0.263 | 0.024* | | 0.122 (-0.337, 0.581) | 0.232 | 0.600 |
| Model 2 | 0.609 (0.132, 1.086) | | 0.242 | 0.013* | | 0.032 (-0.391, 0.455) | 0.214 | 0.882 |
| Model 3 | 0.546 (0.100, 0.991) | | 0.226 | 0.017* | | 0.067 (-0.356, 0.490) | 0.214 | 0.754 |
| Model 4 | 0.087 (-0.214, 0.387) | | 0.153 | 0.570 | | -0.057 (-0.230, 0.116) | 0.088 | 0.515 |
| Model 5 | 0.083 (-0.217, 0.384) | | 0.152 | 0.585 | | -0.051 (-0.235, 0.134) | 0.093 | 0.586 |

Note. SE = robust standard errors to adjust for heteroscedasticity

*p<.05, **p<.01, ***p<.001

Model 1 adjusted for age and grade of employment. Model 2 adjusted for age, grade of employment and waist to hip ratio. Model 3 adjusted for age, grade of employment, waist to hip ratio, smoking, LDL cholesterol, HDL cholesterol, triglycerides, systolic blood pressure and HOMA-IR. Model 4 adjusted for age, grade of employment, waist to hip ratio, smoking, LDL cholesterol, HDL cholesterol, triglycerides, systolic blood pressure, HOMA-IR and baseline levels of pericardial fat. Model 5 adjusted for age, grade of employment, waist to hip ratio, smoking, LDL cholesterol, HDL cholesterol, triglycerides, systolic blood pressure, HOMA-IR, baseline levels of pericardial fat and IL-6.

CI, confidence interval; HDL, high-density lipoprotein; HOMA-IR, insulin resistance; LDL, low-density lipoprotein; IL-6, interleukin-6.

**Supplementary Table 6.** Cross-sectional associations between depressive symptoms and raw values of pericardial fat volume

|  | Males (*N* = 286) | | | | Females (*N* = 188) | | |
| --- | --- | --- | --- | --- | --- | --- | --- |
|  | *B* (95% CI) | SE | | *P* value | *B* (95% CI) | SE | *P* value |
| Model 1 | 1.358 (0.262, 2.454) | 0.557 | 0.015* | | 0.323 (-0.495, 1.141) | 0.415 | 0.437 |
| Model 2 | 1.263 (0.296, 2.231) | 0.492 | 0.011* | | 0.020 (-0.769, 0.808) | 0.400 | 0.961 |
| Model 3 | 1.110 (0.190, 2.029) | 0.467 | 0.018* | | 0.107 (-0.691, 0.905) | 0.404 | 0.791 |
| Model 4 | 0.915 (0.033, 1.796) | 0.448 | 0.042* | | 0.058 (-0.761, 0.877) | 0.415 | 0.889 |

Note. SE = robust standard errors to adjust for heteroscedasticity

*p<.05, **p<.01, ***p<.001

Model 1 adjusted for age and grade of employment. Model 2 adjusted for age, grade of employment and waist to hip ratio. Model 3 adjusted for age, grade of employment, waist to hip ratio, smoking, LDL cholesterol, HDL cholesterol, triglycerides, systolic blood pressure and HOMA-IR. Model 4 adjusted for age, grade of employment, waist to hip ratio, smoking, LDL cholesterol, HDL cholesterol, triglycerides, systolic blood pressure, HOMA-IR and IL-6.

CI, confidence interval; HDL, high-density lipoprotein; HOMA-IR, insulin resistance; LDL, low-density lipoprotein; IL-6, interleukin-6.

**Supplementary Table 7.** Longitudinal associations between depressive symptoms and raw values of pericardial fat volume

|  | Males (*N* = 269) | | | Females (*N* = 181) | | |
| --- | --- | --- | --- | --- | --- | --- |
|  | *B* (95% CI) | SE | *P* value | *B* (95% CI) | SE | *P* value |
| Model 1 | 1.416 (0.258, 2.573) | 0.588 | 0.017* | 0.090 (-0.759, 0.939) | 0.430 | 0.835 |
| Model 2 | 1.357 (0.326, 2.387) | 0.523 | 0.010* | -0.128 (-0.887, 0.631) | 0.385 | 0.740 |
| Model 3 | 1.233 (0.269, 2.197) | 0.490 | 0.012* | -0.055 (-0.802, 0.691) | 0.378 | 0.884 |
| Model 4 | 0.263 (-0.368, 0.893) | 0.320 | 0.413 | -0.219 (-0.542, 0.104) | 0.163 | 0.182 |
| Model 5 | 0.254 (-0.374, 0.883) | 0.319 | 0.427 | -0.209 (-0.545, 0.127) | 0.170 | 0.222 |

Note. SE = robust standard errors to adjust for heteroscedasticity

*p<.05, **p<.01, ***p<.001

Model 1 adjusted for age and grade of employment. Model 2 adjusted for age, grade of employment and waist to hip ratio. Model 3 adjusted for age, grade of employment, waist to hip ratio, smoking, LDL cholesterol, HDL cholesterol, triglycerides, systolic blood pressure and HOMA-IR. Model 4 adjusted for age, grade of employment, waist to hip ratio, smoking, LDL cholesterol, HDL cholesterol, triglycerides, systolic blood pressure, HOMA-IR and baseline levels of pericardial fat. Model 5 adjusted for age, grade of employment, waist to hip ratio, smoking, LDL cholesterol, HDL cholesterol, triglycerides, systolic blood pressure, HOMA-IR, baseline levels of pericardial fat and IL-6.

CI, confidence interval; HDL, high-density lipoprotein; HOMA-IR, insulin resistance; LDL, low-density lipoprotein; IL-6, interleukin-6.
